# Efficacy and safety of transvenous lead extraction using a liberal combined superior and femoral approach

**DOI:** 10.1007/s10840-020-00889-6

**Published:** 2020-10-07

**Authors:** Sing-Chien Yap, Rohit E. Bhagwandien, Dominic A. M. J. Theuns, Yunus Emre Yasar, John de Heide, Mark G. Hoogendijk, Charles Kik, Tamas Szili-Torok

**Affiliations:** 1 Department of Cardiology, Erasmus MC, University Medical Center Rotterdam, Dr. Molewaterplein 40, 3015 GD Rotterdam, the Netherlands; 2 Department of Cardiothoracic Surgery, Erasmus MC, University Medical Center Rotterdam, Rotterdam, the Netherlands

**Keywords:** Implantable cardioverter defibrillator, Infection, Lead failure, Mechanical sheath, Pacemaker, Snare tool, Transvenous lead extraction

## Abstract

**Purpose:**

During transvenous lead extraction (TLE), the femoral snare has mainly been used as a bail-out procedure. The purpose of the present study is to evaluate the efficacy and safety of a TLE approach with a low threshold to use a combined superior and femoral approach.

**Methods:**

This is a single-center observational study including all TLE procedures between 2012 till 2019.

**Results:**

A total of 264 procedures (median age 63 (51–71) years, 67.0% male) were performed in the study period. The main indications for TLE were lead malfunction (67.0%), isolated pocket infection (17.0%) and systemic infection (11.7%). The median dwelling time of the oldest targeted lead was 6.8 (4.0–9.7) years. The techniques used to perform the procedure were the use of a femoral snare only (30%), combined rotational powered sheath and femoral snare (25%), manual traction only (20%), rotational powered sheath only (17%) and locking stylet only (8%). The complete and clinical procedural success rate was 90.2% and 97.7%, respectively, and complete lead removal rate was 94.1% of all targeted leads. The major and minor procedure-related complication rates were 1.1% and 10.2%, respectively. There was one case (0.4%) of emergent sternotomy for management of cardiac avulsion. Furthermore, there were 5 in-hospital non-procedure-related deaths (1.9%), of whom 4 were related to septic shock due to a *Staphylococcus aureus* endocarditis after an uncomplicated TLE with complete removal of all leads.

**Conclusion:**

An effective and safe TLE procedure can be achieved by using the synergy between a superior and femoral approach.

## Introduction

Transvenous lead extraction (TLE) is a technically complex procedure for the removal of indwelling leads and may be associated with serious complications including venous or cardiac perforation requiring emergency surgery. Cardiac implantable electronic device (CIED)-related infections and lead failures are important reasons for TLE. Despite the complexity of the procedure, TLE can be performed successfully using several approaches and tools, including simple manual traction, locking stylets, telescopic sheaths, femoral snares, mechanical powered sheaths and laser sheaths [1–4]. Previous studies have shown that adding femoral snaring (bail-out) to a superior approach increases the complete procedural success rate [5–8]. Some centers prefer femoral snaring as the primary approach with a complete procedural success rate of 94% in experienced centers [9, 10]. However, the femoral approach has been associated with a higher complication and failure rate in comparison to other techniques in the ELECTRa prospective registry [11]. The higher failure rate with the femoral approach in this registry may be biased as the femoral approach is usually used after failure of a superior approach in difficult cases.

Instead of using the femoral snare tool as a bail-out procedure or as a primary approach, we adopted an approach where we used a low threshold to use a femoral snare or a combined superior and femoral approach in order to maximize the complete procedural success rate and to minimize complications. The rational of this approach is to free the lead from encapsulating fibrous or calcified tissue in the axillary-subclavian-brachiocephalic veins with a powered sheath (if necessary); to avoid mechanical dissection with the powered sheath in the superior vena cava (SVC) area to prevent SVC laceration; and to use the benefits of indirect traction (traction applied from an inferior approach) with the femoral snare. The aim of the current study was to assess the efficacy and safety of our approach.

## Methods

### Study population

Using our prospective registry, all TLE procedures in the Erasmus MC (Rotterdam, the Netherlands) between January 2012 till Dec 2019 were evaluated. In the case of CIED-related infection, there was a strong recommendation for early complete device and lead removal. In other cases, the decision to perform a TLE was made on a case-by-case basis after careful discussion with the patient integrating lead (e.g. recall lead, dwell time), procedural (e.g. risk of lead abandonment versus lead extraction) and patient characteristics (e.g. age, comorbidities, pacemaker [PM] dependency, patient preference). The institutional review board of the Erasmus MC approved this study.

### Patient preparation

All TLE procedures were performed in a cardiac catheterization laboratory or hybrid operating room by an experienced lead extraction team, consisting of at least 2 cardiologists, with immediate availability of cardiothoracic surgical backup. Most procedures took place under general anaesthesia, unless the operator decided otherwise based on the anticipated procedural complexity considering the lead dwell time, lead characteristics (e.g. dual-coil ICD lead) and patient characteristics. Anticoagulation was interrupted to minimize the risk of bleeding. A preprocedural venography was performed to identify regions of severe venous stenosis or occlusion and adhesion sites. In patients under general anaesthesia, a preprocedural transesophageal echocardiogram was performed to gain information on lead adhesions, vegetations and pre-existing pericardial effusion. The transesophageal echocardiography probe was left *in situ* to monitor the presence of pericardial effusion during the procedure. Sterile drapings were applied considering the possibility for access for contralateral implantation, emergent pericardiocentesis, thoracentesis, thoracotomy, sternotomy or cardiopulmonary bypass. All patients received invasive hemodynamic monitoring with a radial arterial line. Four units of packed red blood cells were readily available. A “time-out” procedure was performed to prepare the team for the approach of TLE, need for reimplant at the time of extraction, plans for retaining vascular access in case of reimplant and occluded veins and need for temporary pacing. In pacing-dependent patients, a temporary pacing wire was inserted from the femoral or jugular vein.

### Lead extraction approach

In general, we used a stepwise approach for TLE. A horizontal incision was made to permit easy access to the venous entry site. Tissue debridement was performed, especially in patients with pocket infection, and leads were dissected free to their venous entry site with removal of the anchor sleeves. If reimplantation was planned, then ipsilateral venous access was gained and guidewire(s) passed into the SVC if the vein was not occluded. If present, the active-fixation mechanism was retracted, and manual traction was attempted with a standard stylet in place. If lead removal with manual traction was unsuccessful, the lead was cut and a Liberator Beacon tip locking stylet (Cook Medical, Bloomington, IN, USA) and One-Tie Compression Coil (Cook Medical) were placed. The Liberator locking stylet provides focal traction at the tip of the lead and stabilizes the lead. The One-Tie device binds the proximal lead components and locking stylet together.

**Fig. 1:**
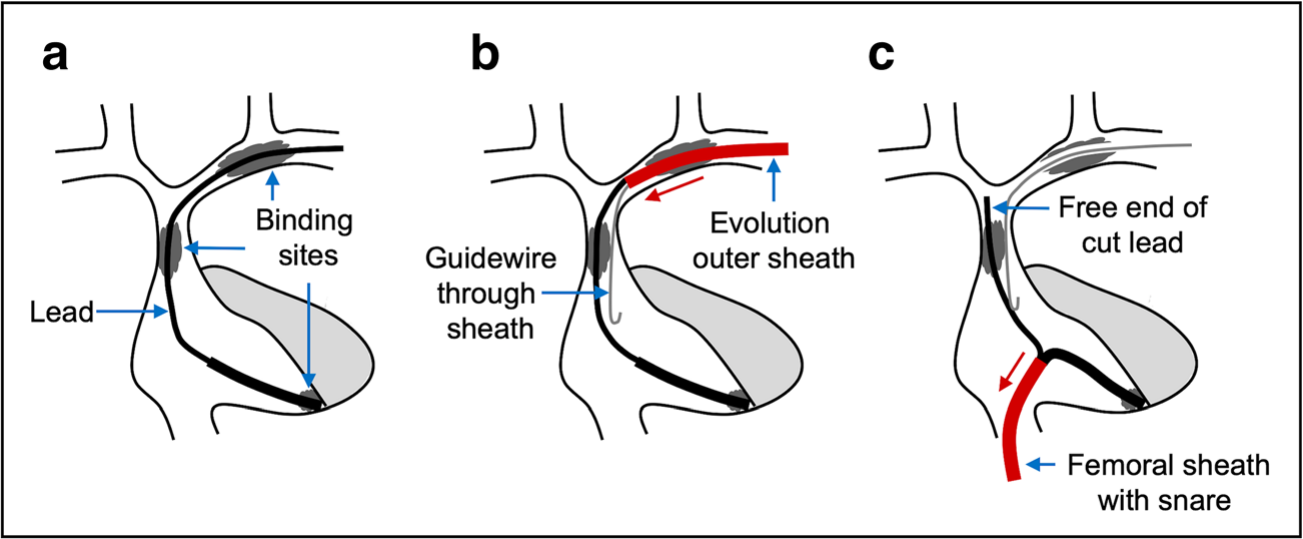
Schematic overview of the combined lead vein entry site and femoral approach. **a** The axillary-subclavian-brachiocephalic veins usually contains the most abundant and resistant encapsulating tissue. **b** In the case of thrombosis of the superior veins or excessive fibrosis, dissection of encapsulating tissue using the Evolution RL or Evolution Shortie RL (Cook Medical, Bloomington, IN, USA) was performed. The powered sheath was advanced over the lead body using counterpressure and countertraction. To reinforce the lead and reduce the risk of lead disruption, the lead was prepared with a Liberator locking stylet (Cook Medical, Bloomington, IN, USA) and One-Tie compression coil (Cook Medical, Bloomington, IN, USA). If needed, a guidewire can be placed through the sheath for maintaining venous access. Mechanical dissection in the SVC area was avoided if possible. **c** Removal of the lead by the femoral work station using a Needle’s Eye Snare (Cook Medical, Bloomington, IN, USA). The 16F outer femoral sheath can be used to perform counterpressure and countertraction. The proximal free end of the cut lead can be pulled down through binding sites in the superior vena cava area

When lead removal with a locking stylet was still unsuccessful, we either proceeded with a mechanical powered sheath or a femoral snare. If resistance was encountered in the superior veins, a mechanical powered sheath was used to dissect the lead from encapsulating fibrous tissue at proximal binding sites (Fig. 1). If no superior binding sites were encountered and venous access could be established in the case of a reimplantation, then a femoral snare was directly used (without the need for a powered sheath).

The rotational mechanism of the hand-powered sheath (11F/13F Evolution and 9F/11F Evolution Shortie, from 2013: Evolution RL and Evolution Shortie RL, Cook Medical) permits movement along the lead body by cutting fibrous or even calcified tissue using the stainless-steel spiral-cut dissection tip. The outer telescoping polymer sheath protects the venous wall from the metal cutting tip while advancing over the lead in tracts free from adhesions. In case of occluded superior veins and the need for reimplantation, we placed a guidewire through the outer sheath after creating a path through the adhesions in the superior veins (Fig. 1b). We avoided mechanical dissection in the area of the SVC to prevent SVC laceration, unless there was a dual-coil shock lead with dense fibrotic adhesions.

If careful continuous steady direct traction fails to extract the lead from the lead vein entry site after freeing the lead from superior binding sites, a Needle’s Eye snare (Cook Medical) was used to extract the lead (Fig. 1c). Thus, we usually do not advance the Evolution sheath up to the tip of the lead. The Needle’s Eye snare has a double loop design which can be used to grasp free-floating lead extremities or the lead body. The 16F Introducer Sheath may not be large enough to accommodate a doubled-up ICD lead, depending on the location of snaring. When necessary, the proximal end of the lead was pulled down to the IVC and the free-floating end was grasped to avoid this issue. Sometimes a simultaneous hybrid superior and femoral approach was used to facilitate extraction and maintenance of vascular access where femoral snaring of the lead stabilizes the lead in order to perform mechanical powered dissection to free the lead and gain vascular access [12].

The timing of device re-implantation, if needed, depended on the indication for TLE, need for ongoing CIED therapy and the complexity of the TLE procedure. Usually, if TLE was performed for lead malfunction, CIED re-implantation was performed during the same procedure. In patients with TLE for CIED-related infection, device re-implantation on the contralateral side was postponed until blood cultures were negative for at least 72 h. In PM-dependent patients with CIED infection, a temporary right ventricular bipolar active fixation lead was implanted through the right jugular vein. The lead was sutured to the patient’s skin with non-resorbable sutures and the lead was connected to a PM generator.

### Definitions

Definitions for procedural approach, techniques, outcomes and complications follow current expert consensus statements [1–3]. Most definitions were initially based on the 2009 HRS expert consensus document on TLE [3]; later expert consensus documents refined the definition of the size of portion of the lead that could be retained to be considered a clinical success [1, 2]. Complete procedural success was defined as removal of all targeted leads and all lead material from the vascular space, with the absence of any permanently disabling complication or procedure-related death. Clinical success was defined as removal of all targeted leads and lead material from the vascular space or retention of a small portion of the lead (< 4 cm) that does not negatively impact the outcome goals of the procedure. A TLE procedure was considered a failure if complete procedural success or clinical success could not be achieved, or if any permanently disabling complication or procedure-related death occurred.

A major complication was defined as any outcome related to the procedure which is life-threatening or results in death. In addition, any unexpected event that causes persistent or significant disability, or any event that requires significant surgical intervention to prevent any of the outcomes listed above is regarded as a major complication. Minor complications are defined as any undesired event related to the procedure that requires medical intervention or minor procedural intervention to remedy, and does not limit persistently or significantly the patient’s function, nor does it threaten life or cause death.

### Statistical analysis

Continuous data are presented as mean ± standard deviation if the data were normally distributed, or as median with interquartile range (25th and 75th percentile) otherwise. Categorical variables are presented by frequencies and percentages. Differences of continuous variables between two groups were analysed with the unpaired Student’s *t* test or the Mann-Whitney *U* test, as appropriate. Differences between categorical variables were evaluated using the Chi-square test or the Fisher’s exact test in case of small expected cell frequencies. Statistical analyses were performed using SPSS V.25.0. All statistical tests were two-sided. *P* values < 0.05 were considered statistically significant.

## Results

### Study population

A total of 264 TLE procedures were performed in the study period. Baseline patient characteristics are presented in Table 1. The median age was 63 (51–71) years and the majority were men (67.0%). Approximately one-fifth (20.8%) of the population had a previous cardiac surgery. The main indications for TLE were lead malfunction (67.0%), isolated pocket infection/erosion (17.0%) and CIED-related systemic infection (11.7%). The median dwelling time of the oldest targeted lead was 6.8 (4.0–9.7) years.

**Table 1:** Baseline characteristics

	*N* = 264
Demographics	
- Age (years), median (IQR)	63 (51–71)
- Male gender	177 (67.0%)
Medical history	
- Coronary artery disease	87 (33.0%)
- Hypertension	65 (24.6%)
- Prior cardiac surgery	55 (20.8%)
- Diabetes mellitus	49 (18.6%)
- Chronic kidney disease (GFR < 45 ml/min)	42 (15.9%)
- Peripheral artery disease	5 (1.9%)
Antithrombotic agents	
- Vitamin K antagonist	108 (40.9%)
- Antiplatelet agent	73 (27.7%)
- NOAC	20 (7.6%)
Device type	
- PM	106 (40.2%)
○ Single-chamber PM^a^	15 (5.7%)
○ Dual-chamber PM	85 (32.2%)
○ Biventricular PM	6 (2.3%)
- ICD	158 (59.8%)
○ Single-chamber ICD^b^	60 (22.7%)
○ Dual-chamber ICD	45 (17.0%)
○ Biventricular ICD	53 (20.1%)
Indications	
- CIED-related infection	70 (26.5%)
○ Isolated pocket infection/erosion	39 (14.8%)
○ Systemic infection	31 (11.7%)
- No CIED-related infection	194 (73.5%)
○ Lead malfunction	169 (64.0%)
○ Other indication^c^	25 (9.5%)
Dwelling time oldest targeted lead (years), median (IQR)	6.8 (4.0–9.7)

Data are presented as number (percentages), unless stated otherwise. *CIED* cardiac implantable electronic device, *ICD* implantable cardioverter defibrillator, *NOAC* non-vitamin K oral anticoagulation, *PM* pacemaker, *TLE* transvenous lead extraction

^a^Including 3 VDD pacemakers

^b^Including 2 VDD ICDs

^c^Including 18 upgrade procedures

In case of lead malfunction, the dysfunctional lead usually comprised an ICD lead (62.1%), followed by an atrial lead (18.6%), right ventricular lead (13.6%) and coronary sinus lead (5.6%). Among 110 dysfunctional ICD leads, the three most common types were Biotronik Linox leads (33.6%), St. Jude Medical Riata leads (30.0%) and St. Jude Medical Durata leads (10.9%).

In 31 patients with CIED-related systemic infection, the most common isolated pathogen was *Staphylococcus aureus* (48.4%). Other pathogens were coagulase-negative staphylococci (12.9%), aerobic Gram-positive nonstaphylococci (12.9%), Gram-negative bacilli (12.9%), anaerobes (3.2%) and *Mycobacterium* species (3.2%). Two patients with a CIED-related systemic infection had negative blood cultures.

### Procedural outcome

**Fig. 2:**
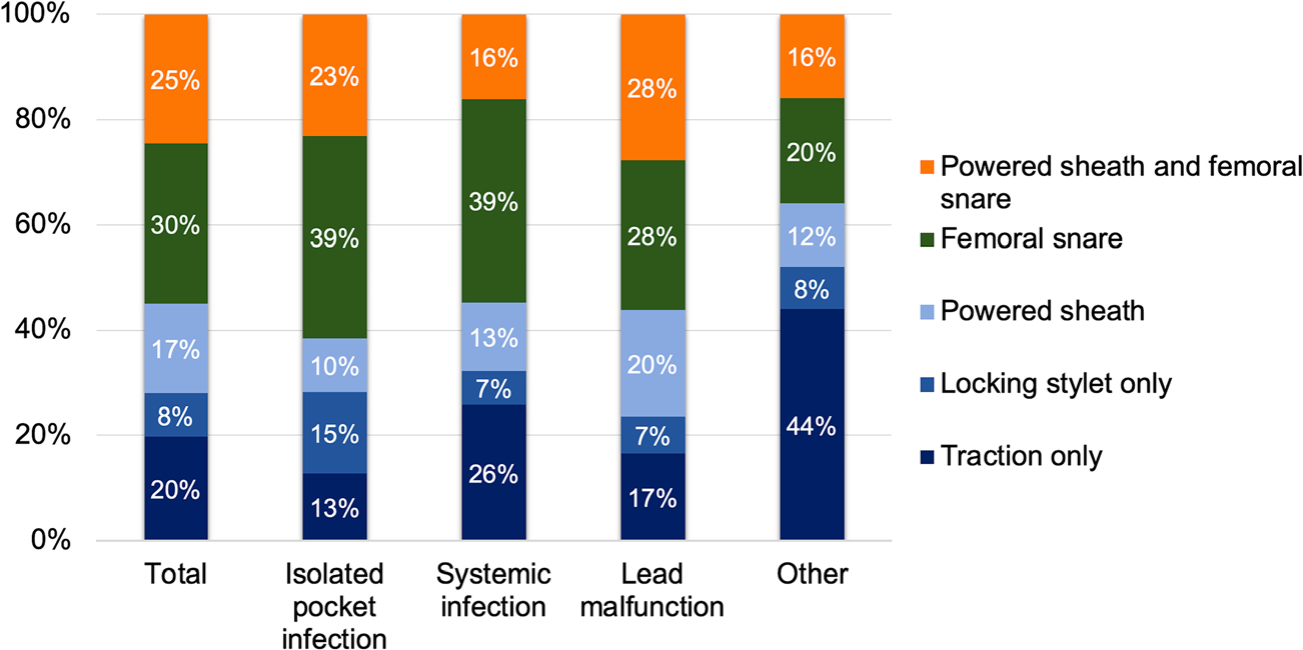
Technique of lead removal for the total group and per indication group. There was a trend towards a relationship between groups with regard to the TLE technique used (*P* = 0.06). The use of the combined superior and femoral approach was numerically the highest in the patients undergoing TLE for lead malfunction

**Fig. 3:**
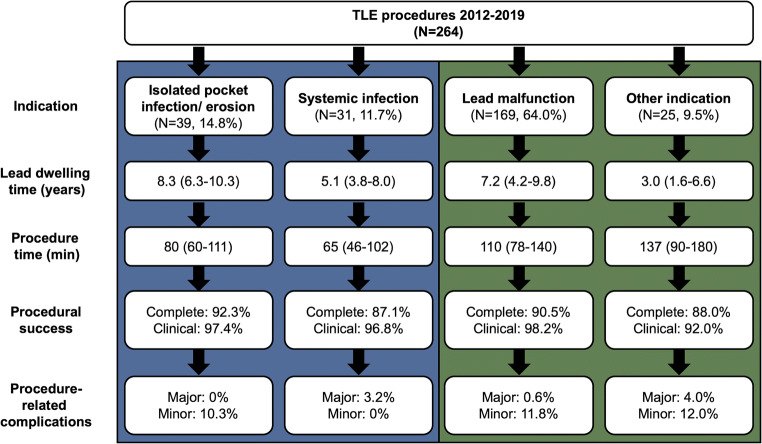
Detailed overview of outcome per indication. There was a difference between the 4 groups with regard to lead dwelling time (*P* < 0.001) and procedure time (*P* < 0.001). After Bonferroni correction, the lead dwelling time in the group “other indication” was shorter in comparison to both the group “lead malfunction” (*P* = 0.002) and the group “isolated pocket infection/erosion” (*P* < 0.001). After Bonferroni correction, there was a difference in procedure time between all groups except between the groups “isolated pocket infection/erosion” and “systemic infection” (*P* = 0.16), and between the groups “lead malfunction” and “other indication” (*P* = 0.08). There were no statistical differences between groups with regard to procedural success and procedure-related complications

An overview of procedural details is presented in Table 2. Most procedures were performed under general anaesthesia. The three most common techniques used to perform the procedure were the use of a femoral snare as a primary tool (30%), combined powered sheath and femoral snare (25%) and traction only (20%) (Fig. 2). Thus, in the majority of the cases (54.5%), a femoral snare was used. The median number of targeted leads were 2 (1–2). The complete procedural success rate and clinical success rate was 90.2% and 97.7%, respectively. Complete lead removal rate was 94.1% of all targeted leads. A detailed overview of the 6 procedural failures is presented in Table 3. An overview of the procedural characteristics and outcome per indication group is presented in Fig. 3.

**Table 2:** Procedural details and outcome

	*N* = 264
General anaesthesia	210 (79.5%)
Extraction tool used:^a^	
- Locking stylet	156 (59.1%)
- Powered sheath	112 (42.4%)
- Femoral snare	144 (54.5%)
Procedure time (min), median (IQR)	102 (70–140)
Fluoroscopy time (min), median (IQR)	12 (7–22)
Leads extracted per case, median (IQR)	2 (1–2)
Procedural outcome	
- Complete procedural success	238 (90.2%)
- Clinical success	258 (97.7%)
- Failure	6 (2.3%)
Reimplantation CIED during same hospital admission	193 (73.1%)
Duration of hospital stay (days), median (IQR)	4 (3–5)
Radiological lead outcome^b^	
- All leads (*N* = 477)	94.1%/4.8%/1.0%
- RA lead (*N* = 158)	96.8%/2.5%/0.6%
- RV lead (*N* = 101)	92.1%/6.9%/1.0%
- CS lead (*N* = 42)	90.5%/9.5%/0%
- ICD lead (*N* = 152)	94.7%/3.9%/1.3%
○ Single coil lead (*N* = 111)^c^	93.7%/5.4%/0.9%
○ Dual coil lead (*N* = 41)^c^	97.6%/0%/2.4%
- Abandoned RA lead (*N* = 7)	100%/0%/0%
- Abandoned RV lead (*N* = 10)	100%/0%/0%
- Abandoned LV lead (*N* = 1)	0%/100%/0%
- Abandoned ICD lead (*N* = 6)	66.7%/16.7%/16.7%

Data are presented as *n* (%), unless stated otherwise. *CIED* cardiac implantable electrical device, *CS* coronary sinus, *ICD* implantable cardioverter-defibrillator, *RA* right atrial, *RV* right ventricular, *TLE* transvenous lead extraction

^a^Different extraction tools could be used in one procedure

^b^Percentages denote complete radiological success, partial radiological success (< 4 cm residual lead portion), and failure, respectively

^c^There was no difference in radiological outcome between single and dual-coil ICD leads (*P* = 0.25)

**Table 3:** Detailed overview of procedural failures

Pt.	Age/sex	Indication TLE	Implanted device	Details
1	36/F	VCS syndrome	Biventricular ICD, abandoned ICD lead	Disruption and breakage of abandoned St. Jude Medical Riata 1582 ICD lead (10 years *in situ*) just proximal to distal coil during indirect traction with snare. Successful SVC stenting.
2	41/M	Upgrade to biventricular ICD	Dual-chamber PM	Wedging and breakage of distal part of Biotronik Solia S ProMRI ventricular lead (2 years *in situ*) at proximal binding site in subclavian vein during direct traction. No attempt with mechanical sheath as new leads were already *in situ*.
3	43/M	ICD and LV lead malfunction	Biventricular ICD	Disruption and breakage of Biotronik Linox Smart S65 ICD lead (5 years *in situ*) just proximal to distal coil during indirect traction with snare.
4	49/F	Fracture of ICD lead	Dual-chamber ICD	Disruption and breakage of Medtronic Sprint Fidelis 6949 ICD lead (10 years *in situ*) distal to proximal coil during indirect traction with snare (after superior approach with powered mechanical sheath failed)
5	62/M	Lead-related endocarditis (*S. epidermidis*)	Dual-chamber PM	Disruption and breakage of tip (< 1 cm) of atrial lead during countertraction with powered mechanical sheath. Complicated by left-sided ischemic stroke the following day with permanent disability (modified Rankin score 3). Most likely due to paradoxical embolus as the patient was known with patent foramen ovale.
6	70/M	Atrial and LV lead malfunction	Biventricular ICD	Wedging of distal part of Biotronik Setrox S53 atrial lead in subclavian vein. Powered mechanical sheath caused excessive bleeding at venous entry site requiring surgical repair, decided to leave remnant lead in place.

*ICD* implantable cardioverter-defibrillator, *PM* pacemaker, *SVC* superior vena cava, *TLE* transvenous lead extraction

Patients who underwent TLE with traction only had a higher complete procedural success rate than patients who required an extraction tool (98.1% versus 88.2%, *P* = 0.03). In contrast, the clinical success rate was similar between patients who underwent TLE with or without extraction tools (97.2% versus 98.1%, respectively, *P* = 1.00). The median dwelling time of the oldest targeted lead was shorter in patients who underwent TLE with traction only (3.3 [1.6–5.2] versus 7.9 [5.1–10.8] years, *P* < 0.001). General anaesthesia was less often used in procedures were TLE was performed with manual traction only (42.3% versus 88.7%, *P* < 0.001).

### Complications

An overview of in-hospital complications is presented in Table 4. The major and minor procedure-related complication rates were 1.1% and 10.2%, respectively. There was one case (0.4%) of emergent sternotomy for cardiac avulsion due to TLE. This was a 57-year-old woman with a dual-chamber ICD who had externalization of her Riata 1580 dual-coil shock lead which was *in situ* for 8 years. An Evolution RL sheath was used to free the lead up to the tricuspid annulus. After this manoeuvre, the patient became hemodynamic unstable and pericardial effusion was drained percutaneously. After complete removal of the ICD lead using the femoral snare, the patient deteriorated despite the drain and an emergent sternotomy was performed demonstrating laceration of the right atrial wall. She recovered clinically and at her last follow-up 5 years later, she is doing well. An overview of the complication rate per indication group is presented in Fig. 3.

**Table 4:** In-hospital complications

	*N* = 264
Procedure-related major complications including deaths	3 (1.1%)
- Stroke^a^	1 (0.4%)
- Cardiac avulsion requiring surgery	1 (0.4%)
- Coronary sinus perforation during reimplantation requiring surgery	1 (0.4%)
Non-procedure-related major complications including deaths	5 (1.9%)
- Death	5 (1.9%)
- Sepsis	4 (1.5%)
- Stroke	1 (0.4%)
Procedure-related minor complications	27 (10.2%)
- Pocket hematoma without intervention	9 (3.4%)
- Pneumothorax requiring a chest tube	3 (1.1%)
- Lead dislocation requiring repositioning	3 (1.1%)
- False aneurysm femoral artery requiring intervention	2 (0.8%)
- Pericardial effusion without intervention	2 (0.8%)
- Pulmonary embolism	2 (0.8%)
- Intra-procedural bleeding requiring blood transfusion	2 (0.8%)
- Migrated lead fragment without sequelae	2 (0.8%)
- Vascular repair at venous entry site	1 (0.4%)
- Pocket hematoma requiring surgical intervention	1 (0.4%)
- Air embolism	1 (0.4%)
- Upper extremity thrombosis	1 (0.4%)
Any procedure-related complication	30 (11.4%)

Data are presented as n (%), unless stated otherwise. Different complications could occur in the same patient

^a^This patient is described in Table 3, patient 5

There were no procedure-related deaths; however, there were 5 in-hospital non-procedure-related deaths after TLE (Table 5). Four patients with *Staphylococcus aureus* CIED-related endocarditis died due to septic shock with multi-organ failure after an uncomplicated complete CIED removal. The interval between diagnosis of CIED-related endocarditis and the TLE procedure was 0, 1, 3 and 9 days. The last patient was presented late to our hospital. Patients who required TLE for a systemic infection had a higher risk of in-hospital nonprocedural-related death in comparison to patients with another indication (12.9% versus 0.4%, *P* = 0.001).

**Table 5:** Overview of in-hospital non-procedural related deaths

Pt.	Age/ sex	Indication TLE	Outcome TLE	Details death
A	55/F	*S. aureus* endocarditis	Complete removal single-chamber ICD	Septic shock with multiorgan failure 2 days after TLE
B	58/M	*S. aureus* endocarditis	Complete removal biventricular ICD	Septic shock with multiorgan failure 1 day after TLE
C	61/M	*S. aureus* endocarditis	Complete removal biventricular ICD	Septic shock with multiorgan failure 2 days after TLE
D	65/M	*S. aureus* endocarditis	Complete removal dual-chamber ICD	Septic shock with multiorgan failure 21 days after TLE
E	89/F^a^	Isolated pocket infection	Complete removal dual-chamber PM	Ischemic stroke 3 days after TLE, thrombolysis followed by thrombectomy, died 9 days after stroke

*ICD*, implantable cardioverter-defibrillator, *PM* pacemaker, *TLE* transvenous lead extraction

^a^Patient had a prior history of atrial fibrillation and recurrent transient ischemic attack

## Discussion

In this study, we evaluated the efficacy and safety of a liberal combined superior and femoral approach for TLE procedures. This approach was associated with a high complete and clinical success rate and a low major complication rate. There were no procedure-related deaths.

### Individualized approach to TLE

TLE has become an integral part of PM and ICD lead management. The number of TLE procedures has increased over the years as a consequence of an increase in CIED implantations, increasing rate of infections, lead failure, and development of extraction tools [2, 13]. An individualized approach is paramount with respect to indication, TLE technique and peri- and post-procedural care for patients undergoing TLE. In clinical practice, a wide spectrum of tools and techniques are used ranging from simple manual traction to combined approaches including powered sheaths and snare tools [11]. In general, the goal of TLE is to achieve the highest clinical success rate with a low complication rate. The most important risk of lead removal includes venous or cardiac perforation requiring emergency surgery. This risk depends on multiple patient and lead-related factors, including the lead dwelling time, lead properties (presence of defibrillator coils, active or passive leads), lead tip location and the presence of prior sternotomy. Although outcomes of TLE has improved as a result of technological advancements in extraction tools, experienced operators and high-volume centers are essential to achieve an optimal TLE outcome [11, 14, 15].

### Role of femoral snaring

Most centers perform a stepwise TLE approach where femoral snaring is used as a bail-out procedure when previous methods have failed [4, 7, 16]. Several single-center studies have shown that adding femoral snaring to a superior approach increases clinical success by approximately 10% [5, 6, 8]. Femoral snaring seems especially useful for older leads and leads with passive fixation which are more prone to fracture [5, 6]. Instead of using femoral snaring as a bail-out procedure, a few single-center studies have demonstrated a high complete procedural success rate (94%) when femoral snaring is used as the primary approach [9, 10]. In these experienced centers, the rate of cardiac tamponade requiring surgical intervention ranged from 0.6 to 0.9%. It is important to note that in these two studies, the proportion of extracted ICD leads was relatively low (0% to 4%) [9, 10], which may positively bias their results as ICD lead removal is known to be associated with a higher risk of major complications [15]. In a European multicenter prospective registry (*n* = 3510), a femoral approach was associated with a higher rate of procedure-related major complications (4.1%), either as primary (9.1%) or secondary (3.5%) approach, compared with other approaches (1.4%) [11]. In addition, the femoral approach was associated with a higher clinical failure rate (odds ratio 3.9) [11]. The higher clinical failure rate may be biased as the femoral approach is usually used as a bail-out procedure in difficult cases. Thus, there is some discrepancy with regard to the procedural outcome of femoral snaring depending of its use as a bail-out procedure or as a primary approach.

### Liberal combined superior and femoral approach

Instead of using femoral snaring as a primary approach or as a bail-out tool, we adopted a novel approach where we used a liberal combined superior and femoral approach or femoral approach only. Major advantages of this approach are (1) maintaining superior venous access in contrast to a strictly femoral approach in case of occluded veins; (2) reducing the resistance on the proximal lead when pulling the lead down from the femoral workstation after freeing the lead from the superior binding sites; (3) avoiding hemodynamic instability by failure of the lead to return to its original position (slippage of lead body through binding site) after direct traction from the vein entry site; and (4) avoiding risk of SVC laceration in contrast to a strictly superior approach [17, 18]. These advantages are especially relevant in case of occluded superior veins. Complete venous occlusion occurs relatively frequently (approximately 10%) after CIED implantation [19], and especially in patients undergoing TLE for device infection (up to 32%) [20]. The complete procedural success rate and clinical success rate in our study was 90.2% and 97.7%, respectively, and complete extraction was achieved for 94.1% of leads (complete lead removal rate).

There was one case (0.4%) of cardiac avulsion requiring emergent surgery, highlighting the relative safety of this approach. This cardiac avulsion occurred in a patient where we had to dissect the dual-coil ICD lead from the SVC using the powered sheath. Despite the use of traction from above to provide a tight “rail”, there was probably an unfavourable sheath-SVC wall geometric relationship creating a RA laceration. Freeing dual-coil ICD leads from severe SVC binding sites may be challenging. For these cases, Schaller et al. have described an interesting technique to reduce the risk of SVC injury by using simultaneous lead traction from above and below (with a femoral snare) during advancement of a powered sheath [21]. Simultaneous traction results in increased separation and a more parallel alignment of the lead and SVC wall, allowing the sheath to be better oriented in the desired lead-vein cleavage plane.

The results of our liberal combined superior and femoral approach are in agreement with the outcomes of high-volume centers (≥ 30 TLE procedures/year) in the ELECTRa registry (TLE procedures between 2012 and 2014) with regard to clinical success rate (97.3%, 95% confidence interval [CI] 96.6–97.8%), complete lead removal rate (96.2%, 95% CI 95.6–96.7%), in-hospital procedure-related major complications (1.5%, 95% CI 1.1–2.0%) and in-hospital procedure-related death (0.4%, 95% CI 0.2–0.7%) [11]. In contrast, our median procedure time was higher than in the ELECTRa registry (102 versus 83 min). This may be related to the relative higher proportion of patients with lead malfunction as these patients require CIED reimplantation during the same procedure (Fig. 3).

Our TLE outcome was also comparable to a recently published European registry, the PROMET (Patient-Related Outcomes of Mechanical lead Extraction Techniques) study, which was focused on the use of rotational TLE tools in 6 high-volume centers [22]. In the PROMET study, clinical success was obtained in 97.0% of procedures (present study 97.7%), and complete lead removal was achieved in 96.5% of targeted leads (present study 94.1%). Whether a liberal combined approach is cost-effective should be further investigated.

Despite similarities in TLE outcomes with 2 large European registries, certain centers have demonstrated a higher complete procedural and clinical success rate [23]. We report our single-center experience with TLE tools from Cook Medical. Perhaps the availability of a wider range of TLE tools (e.g. TightRail, laser sheath) may further improve our TLE results but this should be evaluated.

### Study limitations

This was an observational study without a control group. Therefore, it is difficult to draw firm conclusions whether this approach is better than other techniques. Considering the stepwise approach, selection bias is an issue when comparing the different techniques (superior of femoral approach only versus combined approach) in our study population. In the field of TLE, there is a paucity of randomized controlled trials with regard to comparison between different techniques. Nevertheless, the use of standard definitions of TLE outcome and complications ensures reliable comparison to TLE studies. Finally, the single-center design impacts the generalizability of the data.

## Conclusions

An effective and safe TLE procedure can be achieved by using a stepwise approach with a low threshold to use a combined superior and femoral approach. Thus, instead of using the femoral snare as a last resort, the use of the synergy between a superior and femoral approach may optimize the results of TLE.

## Data Availability

Data are available upon reasonable request to the corresponding author.
